# Neuroprotective Effects of Four Phenylethanoid Glycosides on H_2_O_2_-Induced Apoptosis on PC12 Cells via the Nrf2/ARE Pathway

**DOI:** 10.3390/ijms19041135

**Published:** 2018-04-10

**Authors:** Maiquan Li, Tao Xu, Fei Zhou, Mengmeng Wang, Huaxin Song, Xing Xiao, Baiyi Lu

**Affiliations:** 1 National Engineering Laboratory of Intelligent Food Technology and Equipment, Key Laboratory for Agro-Products Postharvest Handling of Ministry of Agriculture and Rural Affairs, Key Laboratory for Agro-Products Nutritional Evaluation of Ministry of Agriculture and Rural Affairs, Zhejiang Key Laboratory for Agro-Food Processing, Fuli Institute of Food Science, College of Biosystems Engineering and Food Science, Zhejiang University, Hangzhou 310058, China; 11413040@zju.edu.cn (M.L.); txu@zju.edu.cn (T.X.); feizhou@zju.edu.cn (F.Z.); 11613027@zju.edu.cn (M.W.); 21713034@zju.edu.cn (H.S.); 2 College of The First Clinical Medical, Guangzhou University of Chinese Medicine, Guangzhou 510006, China; fushengmengxx@gmail.com

**Keywords:** Keap1, Nrf2, Neuroprotective, PC12 cells, PhGs

## Abstract

Nuclear factor erythroid 2-related factor 2 (Nrf2) is a key transcription factor against oxidative stress and neurodegenerative disorders. Phenylethanoid glycosides (PhGs; salidroside, acteoside, isoacteoside, and echinacoside) exhibit antioxidant and neuroprotective bioactivities. This study was performed to investigate the neuroprotective effect and molecular mechanism of PhGs. PhGs pretreatment significantly suppressed H_2_O_2_-induced cytotoxicity in PC12 cells by triggering the nuclear translocation of Nrf2 and reversing the downregulated protein expression of heme oxygenase 1 (HO-1), NAD(P)H quinone oxidoreductase 1 (NQO1), glutamate cysteine ligase-catalytic subunit (GCLC), and glutamate-cysteine ligase modifier subunit (GCLM). Nrf2 siRNA or HO-1 inhibitor zinc protoporphyrin (ZnPP) reduced the neuroprotective effect. PhGs showed potential interaction with the Nrf2 binding site in Kelch-like ECH-association protein 1 (Keap1). This result may support the hypothesis that PhGs are activators of Nrf2. We demonstrated the potential binding between PhGs and the Keap1-activated Nrf2/ARE pathway, and that PhGs with more glycosides had enhanced effects.

## 1. Introduction

Oxidative stress, which is an imbalance of antioxidant homeostasis, induces lipid peroxidation, injury to protein and DNA, cell aging, and cell death. This process likely contributes to several neurodegenerative disorders, such as Alzheimer’s disease (AD), Parkinson’s disease (PD), and ischemia/reperfusion [1]. Hydrogen peroxide (H_2_O_2_), which is one of the main reactive oxygen species (ROS), is known to cause lipid peroxidation and DNA damage [2]. Moreover, H_2_O_2_ is an endogenous source of hydroxyl free radicals that contributes to the background level of cellular oxidative stress [3,4]. Therefore, therapeutic strategies for preventing oxidative stress-induced apoptosis may have the potential in neurodegenerative diseases treatment.

Nuclear factor erythroid 2-related factor 2 (Nrf2), is a transcription factor that is strongly associated with oxidative stress. Activation of Nrf2 induces the transcription of numerous antioxidant and detoxification genes, including heme oxygenase-1 (HO-1), NAD(P)H quinine oxidoreductase 1 (NQO1), among others [5,6,7]. This process represents a key step in protecting cells from oxidative stress and is emerging as a promising therapeutic target for neuroprotection [8]. Gaia reported that Nrf2 mitigates LRRK2- and α-synuclein-induced neurodegeneration by potently promoting neuronal protein homeostasis in a cell autonomous and time-dependent manner [9].

Phenylethanoid glycosides (PhGs) are characterized by cinnamic acid and hydroxylphenylethyl moieties attached to a β-glucopyranose through ester and glycosidic linkages, respectively. These molecules are members of a group of water-soluble natural products widely distributed in the plant kingdom [10]. In vitro and in vivo studies have shown that these compounds possess antioxidant [11], neuroprotective [12], antibacterial, anti-inflammatory [13], and immunomodulatory [14] bioactivities. *Osmanthus fragrans* is a common ingredient in several Asian foods and has long been consumed. We previously showed that *O. fragrans* flower extracts enhanced spatial learning and memory, inhibited oxidative damage, and exhibited neuroprotective activities in a d-galactose-induced aging in an ICR mouse model [15]. Salidroside, acteoside, and isoacteoside are the major PhGs response for the antioxidant activities of *O. fragrans* flowers extracts [16].

Studies on the neuroprotective effect of PhGs have obtained desirable results. Salidroside significantly reduced cell apoptosis of PC12 cells that was exposed in MPP^+^ [17,18]. Acteoside also alleviated MPP^+^-induced apoptosis and oxidative stress in PC12 cells [19] and Aβ25-35-induced SH-SY5Y cell injury [20]. Echinacoside was investigated on tumor necrosis factor-α (TNFα)-induced apoptosis in SH-SY5Y cells [21], MPTP-induced dopaminergic toxicity in mice [22], glutamate-injured primary cultures of rat cortical cells [23], and 6-OHDA-induced damage in PC12 cells [24]. The results indicated that PhGs exhibited cytoprotective effect and are potential agents to treat neurodegenerative diseases. Studies have shown the antioxidant properties of PhGs underly many other bioactivities for these compounds [25]. However, few studies have investigated the molecular mechanism of PhGs against oxidative toxicity.

In our study, we selected four typical PhGs as follows: salidroside (phenylethanoid monosaccharides), acteoside (phenylethanoid disaccharides), isoacteoside (phenylethanoid disaccharides), and echinacoside (phenylethanoid trisaccharides). We employed a model of neuronal death using differentiated PC12 cells [26] to investigate the protective effect and molecular mechanism of PhGs on H_2_O_2_-induced PC12 cell model. We demonstrated that PhGs activated the Nrf2/ARE pathway by binding to Kelch-like ECH-associated protein 1 (Keap1). This process upregulated the antioxidant enzymes and increased the resistance of PC12 cells to oxidative stress.

## 2. Results

### 2.1. PhGs Suppressed H_2_O_2_-Induced Cytotoxicity in PC12 Cells

**Figure 1:**
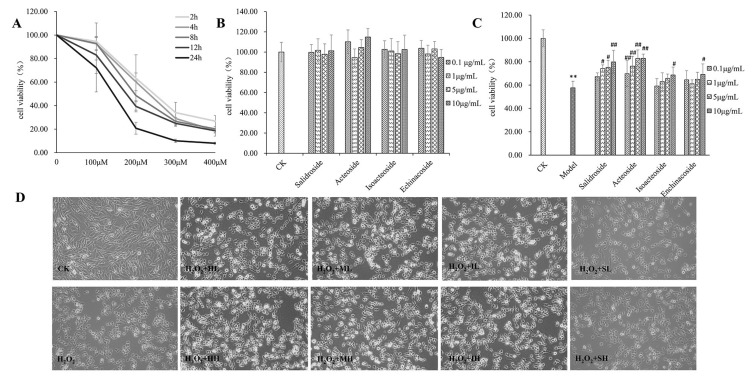
PhGs suppressed H_2_O_2_-induced cytotoxicity in PC12 cells. Cell viability was detected by MTT assay. Cytotoxic effect of H_2_O_2_ (**A**) and PhGs (**B**) at different concentrations on PC12 cells. (**C**) PhGs attenuated H_2_O_2_-induced decrease in cell viability. PC12 cells were incubated with PhGs (0.1, and 10 μg/mL) for 24 h, and then incubated with 200 μM H_2_O_2_ for another 2 h after the PhGs were removed. (**D**) Morphological observation. Cells after treatment were observed by a phase contrast microscope (×100), CK: normal group, H_2_O_2_: H_2_O_2_ treated group, HL: salidroside low dosage treated group, HH: salidroside high dosage treated group, ML: acteoside low dosage treated group, MH: acteoside high dosage treated group, IL: isoacteoside low dosage treated group, IH: isoacteoside high dosage treated group, SL: echinacoside low dosage treated group, SH: echinacoside high dosage treated group. ** *p* < 0.01 versus untreated group; # *p* < 0.05, versus H_2_O_2_ treated group; ## *p* < 0.01, versus H_2_O_2_ treated group.

Cytotoxic effects of H_2_O_2_ and PhGs (0.1, 1, 5, and 10 μg/mL) on PC12 cells were tested. The results showed that H_2_O_2_ induced loss of PC12 cell viability in concentration-dependent and time-dependent manners (Figure 1A). Exposure of PC12 cells to 200 μM H_2_O_2_ for 2 h resulted in cell viability of 57.4%. Pretreatment of cells with PhGs at 0.1, 1, 5, and 10 μg/mL had no effect on cell viability (Figure 1B) and markedly protected PC12 cells from H_2_O_2_-induced damage by improving the cell viability as 9.549–22.141%, 12.092–25.289%, 1.470–9.289%, and 3.411–11.441%, respectively (Figure 1C). However, salidroside (0.1 μg/mL), isoacteoside (0.1, 1, 5, and 10 μg/mL), echinacoside (0.1, 1, and 5 μg/mL) pretreatment showed no significant difference on H_2_O_2_-induced cell injury. Pretreatment of cells with PhGs also ameliorated the morphological characteristic induced by H_2_O_2_ (Figure 1D)_._

### 2.2. PhGs Suppressed H_2_O_2_-Induced Intracellular Accumulation of ROS, Lipid Peroxidation (MDA), and Increased Superoxide Dismutase (SOD) Activities in PC12 Cells

**Figure 2:**
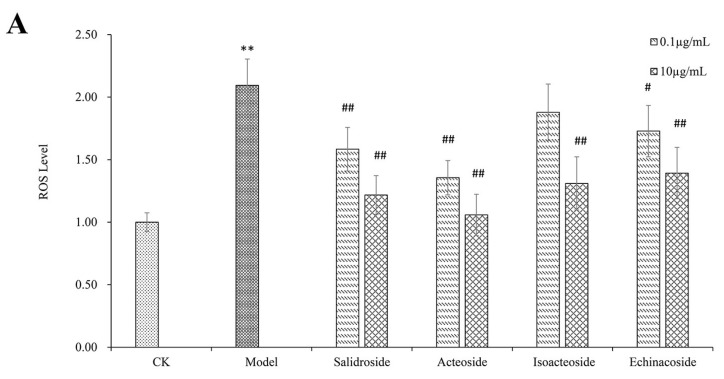

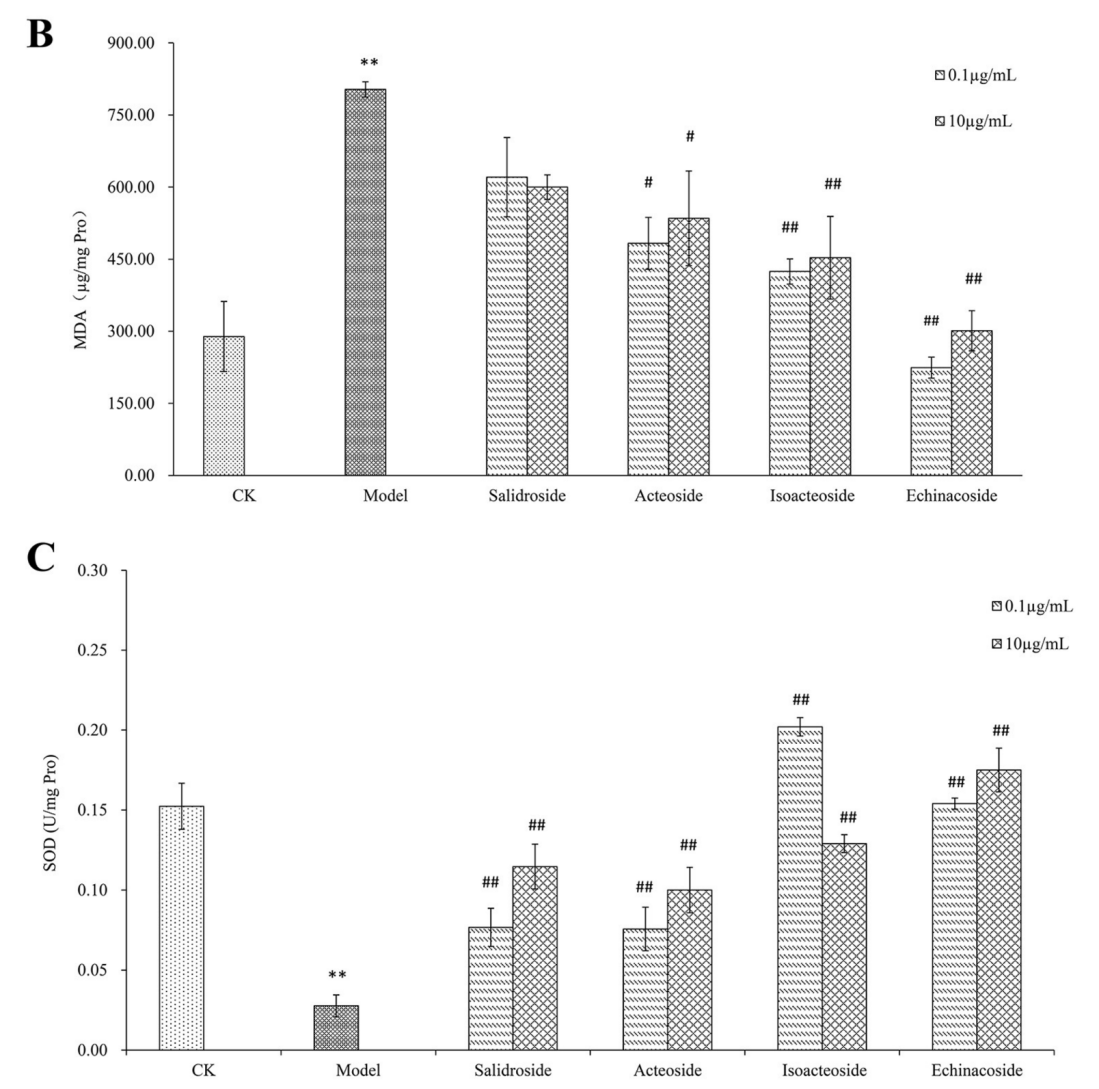
PhGs blocked ROS and MDA accumulation and increased the activities of SOD in PC12 cells. PC12 cells were incubated with PhGs (0.1, and 10 μg/mL) for 24 h, and then incubated with 200 μM H_2_O_2_ for another 2 h after the PhGs were removed. (**A**) PhGs blocked ROS and MDA accumulation. (**B**) PhGs blocked MDA accumulation. (**C**) PhGs increased the activities of SOD. CK: normal group, Model: H_2_O_2_ treated group, Salidroside: salidroside treated group, Acteoside: acteoside treated group, Isoacteoside: isoacteoside treated group, Echinacoside: echinacoside treated group. ** *p* < 0.01 versus untreated group; # *p* < 0.05, versus H_2_O_2_ treated group, ## *p* < 0.01, versus H_2_O_2_ treated group.

Exposure of PC12 cells to 200 μM H_2_O_2_ for 2 h increased ROS levels, MDA content and decreased SOD activity (Figure 2). PhGs pretreatment attenuated ROS level, salidroside, and acteoside, and the high dosage of isoacteoside and echinacoside pretreatment significantly attenuated ROS level (*p* < 0.01). Salidroside pretreatment showed no effect on MDA content, but acteoside pretreatment significantly attenuated MDA content (*p* < 0.05). Isoacteoside and echinacoside pretreatment significantly attenuated MDA content to a greater extent (*p* < 0.01). All PhGs significantly increased SOD activity (*p* < 0.01).

### 2.3. PhGs Reversed H_2_O_2_-Induced Apoptosis in PC12 Cells

**Figure 3:**
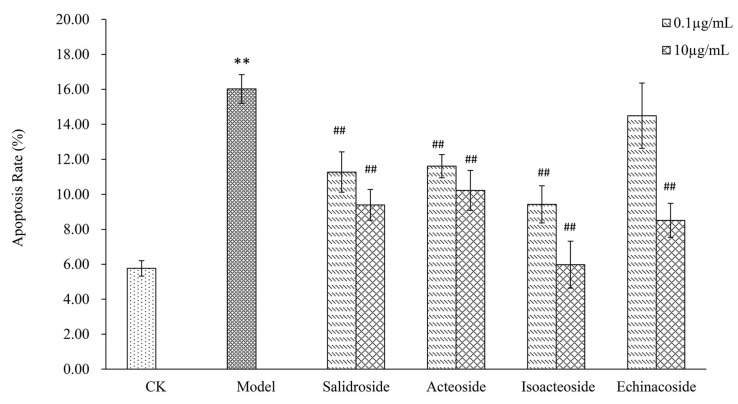
PhGs reversed H_2_O_2_-induced apoptosis in PC12 cells. PC12 cells were incubated with PhGs (0.1, and 10 μg/mL) for 24 h, and then incubated with 200 μM H_2_O_2_ for another 2 h after the PhGs were removed. Then, apoptosis was measured by a flow cytometry using PI/FITC fluorescent probe. CK: normal group, Model: H_2_O_2_ treated group, Salidroside: salidroside treated group, Acteoside: acteoside treated group, Isoacteoside: isoacteoside treated group, Echinacoside: echinacoside treated group. ** *p* < 0.01 versus untreated group; ## *p* < 0.01, versus H_2_O_2_ treated group.

H_2_O_2_ treatment (200 μM) for 2 h significantly increased apoptosis in PC12 cells, with total apoptotic rate up to 16.02% (Figure 3). However, pretreatment with PhGs (0.1 and 10 μg/mL) for 24 h decreased the apoptosis rate in a concentration-dependent manner (*p* < 0.01). Salidroside, acteoside, isoacteoside, and echinacoside markedly decreased the percentage of cell apoptosis by 4.750–6.627%, 4.413–5.800%, 6.593–10.047%, and 1.530–7.510%, respectively.

### 2.4. PhGs Alleviated H_2_O_2_-Induced Dysregulation of the Nrf2-ARE Pathways in PC12 Cells

**Figure 4:**
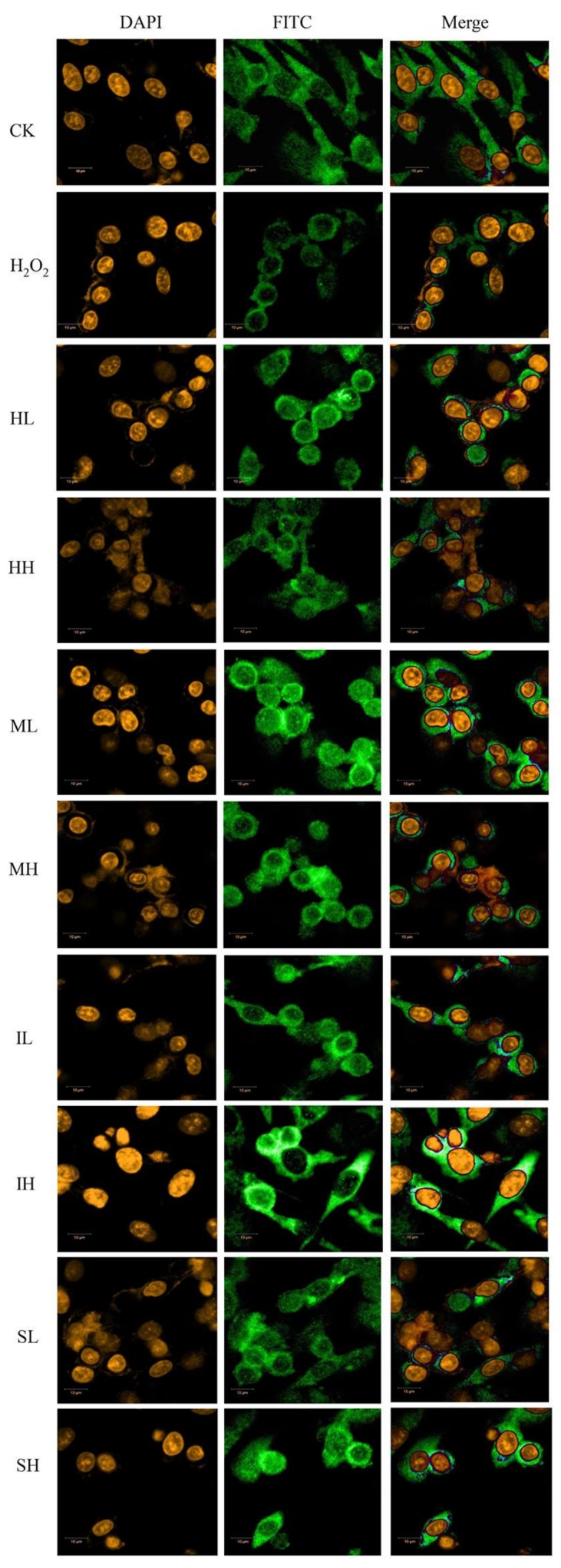
The immunofluorescence assay of Nrf2 in different groups. (×400) PC12 cells were incubated with PhGs (0.1 and 10 μg/mL) for 24 h, and then incubated with 200 μM H_2_O_2_ for another 2 h after the PhGs were removed. Nrf2 localization was observed under an inverted fluorescence microscope. CK: normal group, H_2_O_2_:H_2_O_2_ treated group, HL: 0.1 μg/mL salidroside treated group, HH: 10 μg/mL salidroside treated group, ML: 0.1 μg/mL acteoside treated group, MH 10 μg/mL acteoside treated group, IL: 0.1 μg/mL isoacteoside treated group, IH: 10 μg/mL isoacteoside treated group, SL: 0.1 μg/mL echinacoside treated group, SH: 10 μg/mL echinacoside treated group.

**Figure 5:**
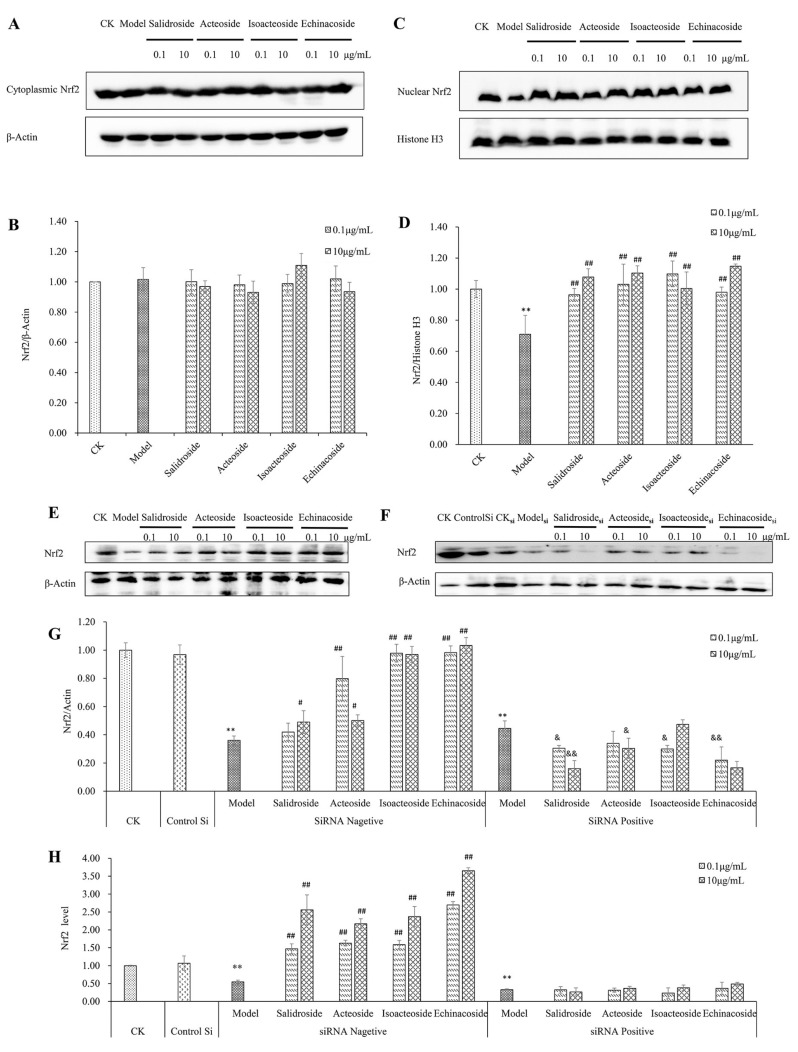

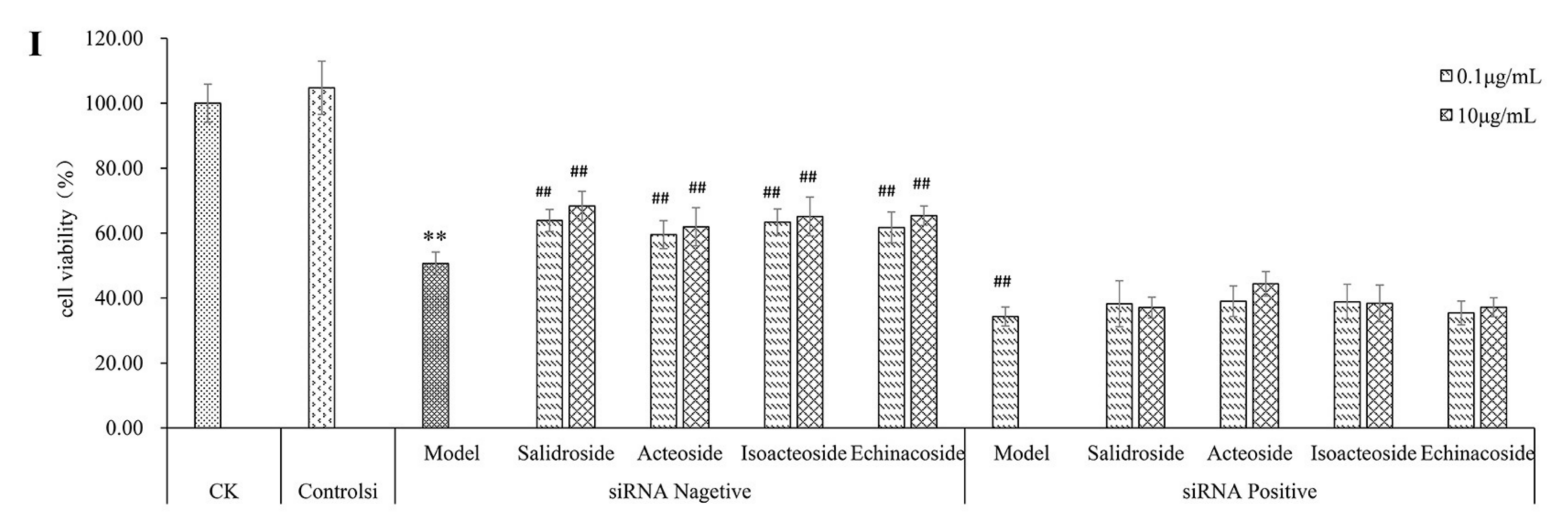
Protective effect of PhGs on Nrf2 in H_2_O_2_-treated PC12 cells. (**A**) The expression of Nrf2 protein in cytoplasm was detected by immunoblotting using specific antibody. β-Actin was used as loading control. (**B**) The quantitative densitometric analysis of Nrf2 protein in cytoplasm. (**C**) The expression of Nrf2 protein in the nucleus was detected by immunoblotting using specific antibody. Histones H3 was used as loading control. (**D**) The quantitative densitometric analysis of Nrf2 protein in the nucleus. (**E**,**F**) PC12 cells were preincubated without (**E**) or with (**F**) Nrf2 siRNA for 24 h, then incubated with or without PhGs (0.1, and 10 μg/mL) for 24 h, and incubated with H_2_O_2_ for another 2 h after the PhGs were removed. Total Nrf2 protein expression was detected by immunoblotting using specific antibody, and β-actin was used as loading control. (**G**) The quantitative densitometric analysis of total Nrf2 protein. (**H**) The quantitative analysis of Nrf2 mRNA. (**I**) PC12 cells were preincubated with or without siRNA for 24 h, then incubated with or without PhGs (0.1 and 10 μg/mL) for 24 h, and incubated with H_2_O_2_ for another 2 h after the PhGs were removed. After treatment, the survival cells were determined by MTT assay. CK: normal group, Model: H_2_O_2_ treated group, ControlSi: control siRNA treated group, SiRNA Negative: without SiRNA treated group, SiRNA Positive: SiRNA treated group, Salidroside: salidroside treated group, Acteoside: acteoside treated group, Isoacteoside: isoacteoside treated group, Echinacoside: echinacoside treated group. ** *p* < 0.01 versus untreated group; # *p* < 0.05, versus H_2_O_2_ treated group (without Nrf2 siRNA treated), ## *p* < 0.01, versus H_2_O_2_ treated group (without Nrf2 siRNA treated); & *p* < 0.05 versus H_2_O_2_ treated group (with Nrf2 siRNA treated), && *p* < 0.01, versus H_2_O_2_ treated group (with Nrf2 siRNA treated).

The Nrf2-ARE pathway, as one of the major antioxidant pathways in most cell type, is important in regulating cell growth and cell death. Therefore, we investigated whether the Nrf2-ARE pathway is involved in H_2_O_2_-induced apoptosis by immunofluorescence and Western blot analysis using specific antibodies. The immunofluorescence assay showed that PhGs (0.1 and 10 μg/mL) induced the nuclear translocation of Nrf2 (Figure 4). After treatment of PhGs, isoacteoside, and echinacoside resulted in the partial recovery of the cell normal morphology. The results from Western blotting and gray density analyses showed that PhGs (0.1 and 10 μg/mL) had no significant influence on the expression of Nrf2 in cytoplasm (Figure 5A,B) (*p* > 0.05) but upregulated Nrf2 expression in the nucleus (Figure 5C,D) (*p* < 0.01). Then, we studied the role of Nrf2 in H_2_O_2_-induced apoptosis by importing the Nrf2 siRNA. The protein and mRNA expression of Nrf2 was significantly downregulated in all Nrf2 siRNA-treated groups (Figure 5E–H). PhGs (0.1 and 10 μg/mL) prevented H_2_O_2_-induced cytotoxicity in groups without Nrf2 siRNA treatment (*p* < 0.01), but such protection was reversed by Nrf2 siRNA. After Nrf2 siRNA treatment, the cell viability decreased significantly even with PhGs treatment (Figure 5I).

### 2.5. PhGs Reversed H_2_O_2_-Induced Downregulation of Protein Expression of HO-1, NQO1, GCLC, and GCLM

**Figure 6:**
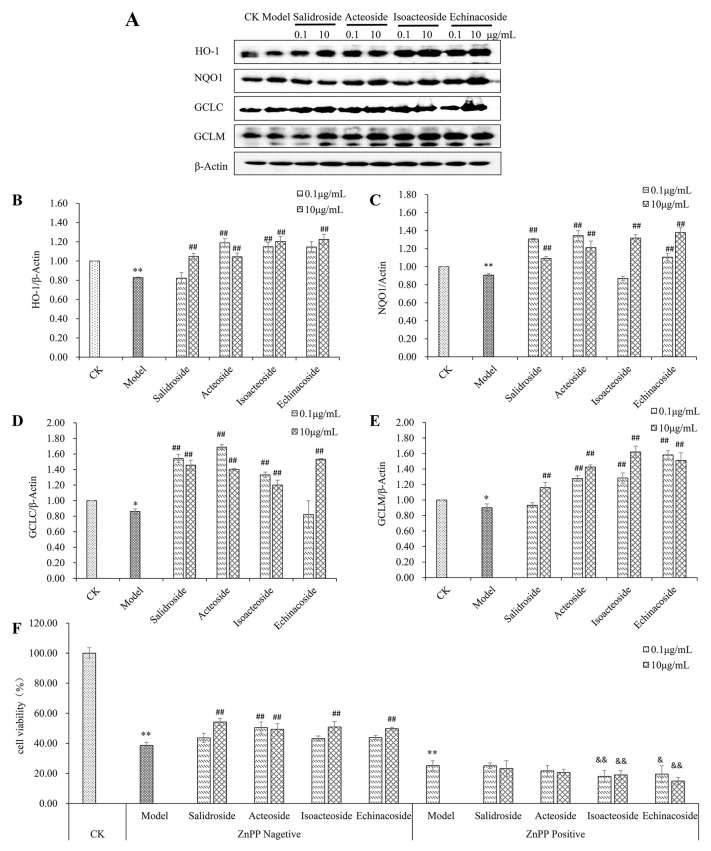
PhGs increased the expression of HO-1, NQO1, GCLC, and GCLM. (**A**) PC12 cells were incubated with PhGs (0.1, and 10 μg/mL) for 24 h, and then incubated with 200 μM H_2_O_2_ for another 2 h after the PhGs were removed. HO-1, NQO1, GCLC, and GCLM protein expression was detected by immunoblotting using specific antibody, and β-actin was used as loading control. (**B**) The quantitative densitometric analysis of HO-1 protein (**C**) The quantitative densitometric analysis of NQO1 protein. (**D**) The quantitative densitometric analysis of GCLC protein. (**E**) The quantitative densitometric analysis of GCLM protein. (**F**) PC12 cells were preincubated with or without ZnPP (20 mM) for 15 min, then incubated with or without PhGs (0.1, and 10 μg/mL) for 24 h, and incubated with H_2_O_2_ for another 2 h after the PhGs were removed. After treatment, the survival cells were determined by MTT assay. CK: normal group, Model: H_2_O_2_ treated group, ZnPP Negative: without ZnPP treated group, ZnPP Positive: ZnPP treated group, Salidroside: salidroside treated group, Acteoside: acteoside treated group, Isoacteoside: isoacteoside treated group, Echinacoside: echinacoside treated group. * *p* < 0.05 versus untreated group, ** *p* < 0.01 versus untreated group; ## *p* < 0.01, versus H_2_O_2_ treated group (without ZnPP treated); & *p* < 0.05, versus H_2_O_2_ treated group (with ZnPP treated), && *p* < 0.01, versus H_2_O_2_ treated group (with ZnPP treated).

HO-1, NQO1, and glutamate-cysteine ligase (GCL) are important cellular antioxidant enzymes, and HO-1, NQO1, and catalytic or modify subunits of GCL (GCLC or GCLM) are Nrf2-regulated downstream genes [27]. Protein expression of HO-1, NQO1, GCLC, and GCLM was observed after treatment. An obvious difference was found between the protein expression of HO-1 and NQO1 with or without H_2_O_2_ (Figure 6A–C) (*p* < 0.01). PhGs (0.1 and 10 μg/mL) reversed the H_2_O_2_-induced downregulation of protein expression of HO-1 (except salidroside at 0.1 μg/mL), NQO1 (except acteoside at 0.1 μg/mL) (*p* < 0.01). H_2_O_2_ also downregulated GCLC and GCLM protein expression (*p* < 0.05) (Figure 6A,D,E). PhGs (0.1 and 10 μg/mL) reversed H_2_O_2_-induced downregulation of protein expression of GCLC (except echinacoside at 0.1 μg/mL) (*p* < 0.01) and GCLM (except salidroside at 0.1 μg/mL) (*p* < 0.01). Then, the chemical inhibitors for HO-1 were used to further evaluate the roles of the antioxidant enzymes in regulating the protection of PhGs against H_2_O_2_-induced cytotoxicity. PhGs (0.1 and 10 μg/mL) prevented H_2_O_2_-induced cytotoxicity, but such protective effect was reversed by HO-1 inhibitor ZnPP (*p* < 0.01) at 20 μM (Figure 6F, *p* < 0.01).

### 2.6. Keap1 Expression and Molecular Docking Analysis

**Figure 7:**
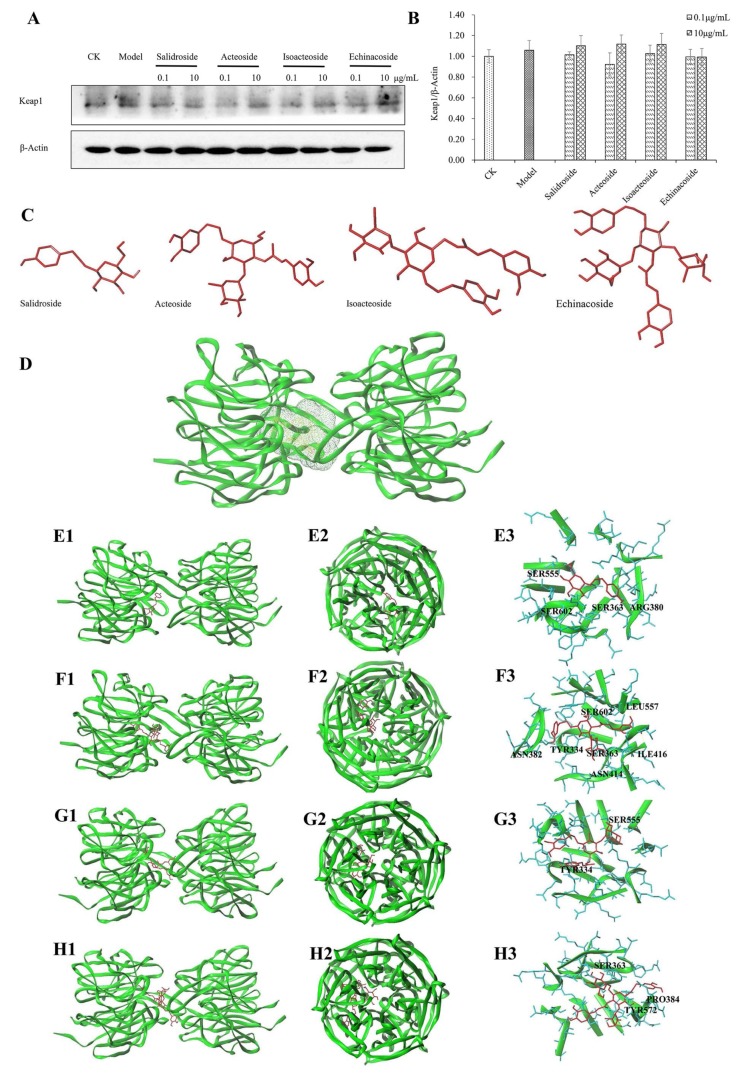
Effect of PhGs on Keap1 expression and molecular docking results. (**A**) PC12 cells were incubated with PhGs (0.1, and 10 μg/mL) for 24 h, and then incubated with 200 μM H_2_O_2_ for another 2 h after the PhGs were removed. Keap1 protein expression was detected by Western blotting using specific antibody, and β-actin was used as loading control. (**B**) The quantitative densitometric analysis of Keap1 protein. (**C**) 3D structure of salidroside, acteoside, isoacteoside, and echinacoside (**D**) The binding pocket of Keap1. (**E1**–**E3**) Representative amino acid residues surrounding salidroside (red) in the binding pocket of Keap1 (**E1** front view, **E2** side view, **E3** binding sites). (**F1**–**F3**) Representative amino acid residues surrounding acteoside (red) in the binding pocket of Keap1 (**F1** front view, **F2** side view, **F3** binding sites). (**G1**–**G3**) Representative amino acid residues surrounding isoacteoside (red) in the binding pocket of Keap1 (**G1** front view, **G2** side view, **G3** binding sites). (**H1**–**H3**) Representative amino acid residues surrounding echinacoside (red) in the binding pocket of Keap1 (**H1** front view, **H2** side view, **H3** binding sites). The dotted line (yellow) indicate potential interactions between amino acid residues and PhGs.

Under physiological conditions, Keap1 acts as a repressor protein of Nrf2 by binding to the Neh2 domain of Nrf2 and targeting Nrf2 to a Cul3-based E3 ubiquitin ligase for ubiquitination and subsequent degradation by the 26S proteasome [28]. Binding capacity to Keap1 of PhGs was evaluated by molecular docking analysis to investigate the mechanism under their antioxidant effect. The expression of Keap1 protein did not change before and after PhGs treatment (Figure 7A,B). Molecular docking results showed that the Keap1 protein domain had a relatively active binding pocket (Figure 7D), and PhGs could bind to Keap1 (Figure 7E–H). Analytical results showed that salidroside exhibited relatively weaker binding capacity. C score ≥ 4 and Total-score > 6 of echinacoside, isoacteoside, and acteoside indicated their better binding capacity with Keap1 (Table 1).

## 3. Discussion

We investigated the neuroprotection of PhGs on H_2_O_2_ induced-cytotoxicity in PC12 cells. The results show that PhGs pretreatment significantly suppressed H_2_O_2_-induced cytotoxicity, attenuated the intracellular ROS level, improved the level of intracellular antioxidant enzymes, and ultimately reversed H_2_O_2_-induced cytotoxicity in PC12 cells. Moreover, PhGs increased the transcriptional activation of Nrf2, reversed the H_2_O_2_-induced downregulation of the protein expression of HO-1, NQO1, GCLC, and GCLM. In addition, PhGs showed potential interaction with Nrf2 binding site in the Keap1 protein.

In the H_2_O_2_-induced PC12 cell injury, lipid peroxidation, which refers to oxidative degradation of lipid, increased the permeability of membranes, leading to cell damage [29]. MDA formation is widely used as the index of lipid peroxidation [30]. H_2_O_2_ enhanced ROS production and exhausted antioxidant defense enzymes, such as SOD, catalase, and GPx. This process leads to oxidative stress [31], which plays a key role in the causation and progression of the majority of neurodegenerative disorders. Consistent with previous studies, we observed an increased level of ROS, reduced intracellular antioxidant enzymes, and enhanced apoptosis of PC12 cells after H_2_O_2_ treatment. PhGs pretreatment significantly attenuated H_2_O_2_-induced increase in intracellular ROS, improved intracellular antioxidant enzymes, and ultimately reversed H_2_O_2_-induced cytotoxicity in PC12 cells.

Kuang et al. [32] reported that echinacoside showed significant neuroprotective effect on H_2_O_2_-induced cytotoxicity in PC12 cells through the mitochondrial apoptotic pathway. In this study, we found that echinacoside, salidroside, acteoside, and isoacteoside showed neuroprotective effect by enhancing the antioxidant activity of PC12 cells because they increased the transcriptional activation of Nrf2 and upregulated the downstream protein expression of HO-1, NQO1, GCLC, and GCLM. Numerous studies have clearly demonstrated that activation of Nrf2 target genes, in particular HO-1, in astrocytes and neurons strongly protect against inflammation, oxidative damage, and cell death. The HO-1 system has been reported to be very active in the central nervous system, and its modulation apparently plays a crucial role in the pathogenesis of neurodegenerative disorders [33]. Recent studies also clarified the role of Nrf2 in the progression and risk of PD [9] and Keap1 as an efficient target for the reactivation of Nrf2 in AD [34]. The results support new evidence for Nrf2 as a therapeutic target in neurodegenerative diseases.

Molecular docking analysis showed that PhGs could bind to Keap1, with the following binding capacities: echinacoside > isoacteoside > acteoside > salidroside. Consistent with these results, PhGs pretreatment led to the Nrf2 nuclear translocation, with the following Nrf2 expression in the nucleus: echinacoside > isoacteoside ≈ acteoside > salidroside. We assumed that the number of glycosides affected the possible binding mode of PhGs and Keap1, and the binding mode further caused the release of Nrf2 from Keap1. This process resulted in the activation of Nrf2 and the downstream genes and ultimately protect PC12 cells from H_2_O_2_-induced oxidative stress.

In summary, PhGs with more glycosides showed an enhanced effect on Nrf2 activation. PhGs induced Nrf2 activation by blocking the binding between Nrf2 and Keap1. This process activated the Nrf2/ARE pathway and protected PC12 from H_2_O_2_-induced cytotoxicity.

## 4. Materials and Methods 

### 4.1. Chemical Compounds and Reagents

Salidroside (CAS No. 10338-51-9), acteoside (CAS No. 61276-17-3), isoacteoside (CAS No. 61303-13-7), and echinacoside (CAS No. 82854-37-3) were purchased from Yuanye Biotechnology Company (Shanghai, China). The PhGs were dissolved in PBS to produce a 10 mg/mL stock solution, which was stored at −20 °C. H_2_O_2_ was purchased from Aladdin^®^ (Shanghai, China). RPMI-1640 medium and fetal bovine serum were purchased from Hyclone (Logan, UT, USA), and 0.5% trypsin EDTA, penicillin, and streptomycin were purchased from Keyi (Hangzhou, China). MDA, SOD diagnostic kits, MTT, and DCFH-DA were purchased from Beyotime Institute of Biotechnology (Nanjing, Jiangsu, China). Annexin V-FITC/PI double staining Kit was purchased from Solarbio Life Sciences (Beijing, China). Antibodies to Nrf2, Histone H3, Keap1, HO-1, NQO1, GCLC, GCLM, and β-actin, anti-mouse-horseradish peroxide (HRP) IgG, and anti-rabbit-HRP-IgG were purchased from Abcam (London, UK). Inhibitors of HO-1 and ZnPP were purchased from Sigma Chemical Co. (St. Louis, MO, USA). RNAiso Plus, PrimeScript™RT reagent Kit with gDNA Eraser, and SYBR^®^ Premix Ex Taq™ II were bought from Takara (Shiga, Japan). Lipofectamine^®^ RNAiMAX Transfection Reagent was purchased from Thermo Fisher Scientific (Waltham, UK). The Nrf2 siRNA sequences were as follows: forward, CCGAAUUACAGUGUCUUAA; and reverse, UUAAGACACUGUAAUUCGG. Meanwhile, control siRNA sequences were as follows: forward, UUCUCCGAACGUGUCACGU; and reverse, ACGUGACACGUUCGGAGAA.

### 4.2. Cell Culture

Mouse adrenal pheochromocytoma line (PC12 cells) was obtained from the Institute of Biochemistry and Cell Biology, SIBS, (CAS, Shanghai, China). The cells were maintained in RPMI-1640 (Hyclone) containing 10% fetal bovine serum (Hyclone), 100 U/mL penicillin, and 0.1 mg/mL streptomycin at 37 °C with 5% CO_2_. The medium was changed every other day.

### 4.3. Cell Viability Assay

PC12 cells were seeded in 96-well plates at 2 × 10^4^ cells/well. After attachment, cells were preincubated with or without inhibitor for 20 min, incubated with or without PhGs for 24 h, and then incubated with H_2_O_2_ for another 2 h after the PhGs were removed. After incubation, the cells were treated with 5 mg/mL MTT for 4 h at 37 °C, and the media were carefully removed. The formazan crystals that had formed by surviving cells were dissolved in 150 μL of DMSO to generate a blue color [35], and the absorbance was measured at 570 nm on a plate reader. Controls utilized the same concentration of medium with DMSO alone. Cell viability was normalized as the percentage of control.

The concentrations of PhGs (0.1, 1, 5, and 10 μg/mL) was chosen depending on the cytotoxicity analysis of PhGs and the reported cytoprotective effect of echinacoside [32]. According to the report there was no cytotoxicity effect shown below 10 μg/mL, and echinacoside was reported to show cytoprotective effect in H_2_O_2_-injured cell model.

### 4.4. Apoptosis Assay

Apoptosis was detected with an Annexin V-FITC/PI double staining Kit (Solarbio). PC12 cells were seeded in 6-well plates at 2 × 10^5^ cells/well. After attachment, cells were treated with PhGs (0.1 and 10 μg/mL) for 24 h and incubated with H_2_O_2_ for another 2 h after the PhGs were removed. After incubation, the cells were washed in cold PBS, centrifuged twice at 1500 rpm for 10 min, and resuspended in 500 μL of binding buffer. FITC-labeled Annexin V (5 μL) and propidium iodide (PI, 5 μL) were then added to cells. Then, the cells were incubated in the dark at room temperature for 20 min according to the manufacturer’s instruction. Cell apoptosis was measured using a Gallios™ flow cytometer (Beckman Coulter, Brea, CA, USA). Annexin V-positive and PI-negative cells were scored as early apoptotic cells, while cells double-stained with both Annexin V and PI were considered as late apoptotic cells. Control cells were negative for both stains.

### 4.5. Measurement of Intracellular ROS, MDA Production, and SOD

The intracellular ROS level was determined using a ROS-sensitive fluorescent probe, namely, 2,7-dichlorodihydro fluorescent diacetate (DCFH-DA). PC12 cells were seeded in 6-well plates at 2 × 10^5^ cells/well. After attachment, cells were treated with PhGs (0.1 and 10 μg/mL) for 24 h and incubated with H_2_O_2_ for another 2 h after the PhGs were removed. After incubation, the cells were washed with PBS thrice and subsequently incubated with 10 µM DCFH-DA. After incubation at 37 °C for 20 min, the cells were washed with PBS and collected by gentle centrifugation. Intracellular ROS was measured using a Gallios™ flow cytometer (Beckman Coulter, Brea, CA, USA).

MDA and SOD were measured by assay kits (Beyotime Biotechnology, Nanjing, Jiangsu, China). All procedures completely complied with the manufacturers’ instructions. The contents of MDA and SOD were normalized with the corresponding total protein content.

### 4.6. Nrf2 Nuclear Translocation Immunofluorescence

The PC12 cells were seeded in 6-well glass slides at a density of 2 × 10^5^ per well. After attachment, the cells were treated with PhGs (0.1 and 10 μg/mL) for 24 h and incubated with H_2_O_2_ for another 2 h after the PhGs were removed. After incubation, the cells were washed in cold PBS and fixed in 4% paraformaldehyde for 15 min at room temperature, followed by membrane permeabilization using 0.5% Triton X-100 in PBS for 5 min. The cells were incubated with anti-Nrf2 Rabbit IgG (Abcam) with 5% FBS overnight at 4 °C. Then, the secondary anti-rabbit antibodies conjugated with FITC were applied to the cells for 20 min at room temperature. The nuclei were stained with 4′,6-diamidino-2-phenylindole (DAPI) at the final preparation step. The slides were rinsed briefly with PBS, air-dried, and mounted in an anti-fluorescence in fading medium. The slides were visualized under a laser confocal microscope (LMS780, Zeiss, Germany).

### 4.7. Protein Extraction

The PC12 cells were seeded in 100 mm dishes at 1 × 10^7^ cells/dish. After attachment, the cells were treated with PhGs (0.1 and 10 μg/mL) for 24 h and then incubated with H_2_O_2_ for another 2 h with PhGs removal. After incubation, the cells were washed twice with cold PBS and scraped from the dishes with 1000 µL of PBS. Cell homogenates were centrifuged at 1500 rpm for 10 min. After treatment, cellular proteins were extracted using a Beyotime RIPA cell lysis buffer with 1 mM PMSF according to the manufacturer’s instructions. In addition, cytoplasmic and nuclear proteins were isolated as described in the Beyotime nuclear and cytoplasmic extraction kit. Protein concentration of the samples was detected by a Beyotime BCA protein assay kit, and all samples were stored at −80 °C for Western blot analysis.

### 4.8. Western Blot Analysis

Western blot analysis was performed using standard methods. Briefly, protein samples (20 or 30 µg) were separated by SDS-PAGE and transferred to PVDF membranes. Membranes were blocked in 5% milk-TBST and incubated overnight at 4 °C in primary antibody. Antibodies used included Nrf2 (Abcam), Keap1 (Abcam), HO-1 (Abcam), NQO-1 (Abcam), GCLC (Abcam), GCLM (Abcam), Histone H3 (Abcam), and β-actin (Abcam). Peroxidase-conjugated anti-mouse IgG (Abcam) or anti-rabbit IgG (Abcam) was used as the secondary antibody. The protein bands were visualized using ChemiScope series (Clinx Science Instruments, Shanghai, China). Gray value of protein bands was quantified using ImageJ (National Institutes of Health, Bethesda, MD, USA).

### 4.9. Quantitative Real-Time PCR

Total RNA was extracted using the RNAiso Plus (Takara, Shiga, Japan), following the manufacturer’s instructions. Total RNA samples were reverse transcribed with PrimeScript™RT reagent Kit with gDNA Eraser (Takara, Shiga, Japan) according to the manufacturer’s instruction. Quantitative real-time PCR was performed using SYBR^®^ Premix Ex Taq™ II (Takara, Shiga, Japan) in Applied Biosystems ViiA™ 7 Real-Time PCR System. Relative expression of target genes was normalized to β-Actin and analyzed by 2^−ΔΔ*C*t^ method. Primer sequences for Nrf2 were as follows: forward, ACAGTGCTCCTATGCGTGAA and reverse, TCTGGGCGGCGACTTTAT. Primer sequences for β-Actin were as follows: forward, GCTGTCCCTGTATGCCTCT; and reverse, TTGATGTCACGCACGATTT.

### 4.10. siRNA Transfection

PC12 cells were cultured in 6-well glass slides at a density of 2 × 10^5^ per well (for Western blot analysis) or in 96-well glass slides at a density of 2 × 10^4^ per well (for cell viability analysis). Control siRNA and Nrf2 siRNA were transfected into cells using Lipofectamine RNAiMAX (Thermo Fisher Scientific, Waltham, MA, USA) according to the manufacturer’s instructions. After 24 h of incubation, the cells were treated with or without PhGs and H_2_O_2_ for the indicated times and then used in Western blot analysis, qRT-PCR, or cell viability analysis as described above.

### 4.11. Molecular Docking Analysis

Molecular docking analysis was performed to investigate the possible binding mode of PhGs to Keap1. The 3D structure of ligands was obtained from NCBI. The crystalized structure of Keap1 (PDB Code: 4L7B) was prepared using correcting structure issues (such as break bond and miss loop) using SYBYL-X 2.0. In this software simulation, crash represents the inadequate penetration between the protein and the ligand. Polar represents the hydrogen bonding and electrostatic interactions between the protein and the ligand. G-score [36] represents the hydrogen bonding, complex (ligand–protein), and internal (ligand–ligand) energies between the protein and the ligand. PMF-score [37] represents the Helmholtz free energy between the protein and the ligand. D-score [38] represents the charge and van der Waals interactions between the protein and the ligand. Chem-score [39] represents the hydrogen bonding, metal-ligand interaction, lipophilic contact, and rotational entropy, along with an intercept term between the protein and the ligand. C score represents the consensus score between the protein and the ligand, comprehensively reflecting the G, PMF, D, and Chem-scores. Tota-score reflects the binding capacity of ligand to protein. The best modes were generated and evaluated using the Total-score (>6) and C-score (≥4).

### 4.12. Statistical Analysis

Data were analyzed using one-way ANOVA with LSD analyses using SPSS. All results were confirmed from three independent experiments. Data were expressed as means ± SD. Statistically significant differences were considered at *p* < 0.05.

## Figures and Tables

**Table 1:** Molecular docking analysis of PhGs.

Compounds Name	Total-Score	Crash	Polar	D-Score	PMF-Score	G-Score	Chem-Score	C-Score
Salidroside	5.0951	−1.0622	5.1831	−561.448	−30.2413	−115.923	−22.6459	4
Acteoside	7.4971	−2.3674	7.8899	−1562.04	−67.978	-192.18	−33.947	5
Isoacteoside	8.1776	−1.4	6.4762	−1439.24	−88.9989	−155.055	−26.1735	4
Echinacoside	9.3402	−2.6794	9.6386	−1756.32	−82.9345	−187.808	−32.4387	4

Crash: The inadequate penetration between the protein and the ligand. Polar: The hydrogen bonding and electrostatic interactions between the protein and the ligand. D-score: The charge and van der Waals interactions between the protein and the ligand. PMF-score: The helmholtz free energy between the protein and the ligand. G-score: The hydrogen bonding, complex (ligand-protein), and internal (ligand-ligand) energies between the protein and the ligand. Chem-score: The hydrogen bonding, metal-ligand interaction, lipophilic contact, and rotational entropy, along with an intercept term between the protein and the ligand. C-score: The concensus score between the protein and the ligand, comprehensive reflecting the D-score, PMF-score, G-score, and Chem-score.

## Abbreviations

GCLC	glutamate cysteine ligase-catalytic subunit
GCLM	glutamate cysteine ligase-catalytic modifier subunit
H_2_O_2_	hydrogen peroxide
HO-1	heme oxygenase 1
Keap1	Kelch ECH association protein 1
NQO1	NAD(P)H quinone oxidoreductase 1
Nrf2	nuclear factor erythroid 2-related factor 2
PhGs	salidroside, acteoside, isoacteoside, and echinacoside
ROS	reactive oxygen species
ZnPP	zinc protoporphyrin
